# Role of aryl hydrocarbon receptors in infection and inflammation

**DOI:** 10.3389/fimmu.2024.1367734

**Published:** 2024-04-12

**Authors:** Linglan Xu, Luping Lin, Nan Xie, Weiwei Chen, Weihua Nong, Ranhui Li

**Affiliations:** ^1^ Key Laboratory of Research on Clinical Molecular Diagnosis for High Incidence Diseases in Western Guangxi, Department of Obstetrics and Gynecology, Affiliated Hospital of Youjiang Medical University for Nationalities, Baise, Guangxi, China; ^2^ Hunan Provincial Key Laboratory for Special Pathogens Prevention and Control, Institute of Pathogenic Biology, Hengyang Medical School, University of South China, Hengyang, China; ^3^ Hunan Prevention and Treatment Institute for Occupational Diseases and Affiliated Prevention and Treatment Institute for Occupational Diseases, University of South China, Changsha, China

**Keywords:** AhR, viruses, bacteria, parasites, fungus

## Abstract

The aryl hydrocarbon receptor (AhR) is a transcription factor that is activated by various ligands, including pollutants, microorganisms, and metabolic substances. It is expressed extensively in pulmonary and intestinal epithelial cells, where it contributes to barrier defense. The expression of AhR is pivotal in regulating the inflammatory response to microorganisms. However, dysregulated AhR expression can result in endocrine disorders, leading to immunotoxicity and potentially promoting the development of carcinoma. This review focuses on the crucial role of the AhR in facilitating and limiting the proliferation of pathogens, specifically in relation to the host cell type and the species of etiological agents involved in microbial pathogen infections. The activation of AhR is enhanced through the IDO1-AhR-IDO1 positive feedback loop, which is manipulated by viruses. AhR primarily promotes the infection of SARS-CoV-2 by inducing the expression of angiotensin-converting enzyme 2 (ACE2) and the secretion of pro-inflammatory cytokines. AhR also plays a significant role in regulating various types of T-cells, including CD4^+^ T cells and CD8^+^ T cells, in the context of pulmonary infections. The AhR pathway plays a crucial role in regulating immune responses within the respiratory and intestinal barriers when they are invaded by viruses, bacteria, parasites, and fungi. Additionally, we propose that targeting the agonist and antagonist of AhR signaling pathways could serve as a promising therapeutic approach for combating pathogen infections, especially in light of the growing prevalence of drug resistance to multiple antibiotics.

## Introduction

The aryl hydrocarbon receptor (AhR) belongs to the basic helix-loop-helix (bHLH) family and functions as a transcription factor that is activated through binding with its cognate ligand. This activation is involved in regulating host immune homeostasis and metabolism (1). Since its discovery by Alan Poland in the mid-1970s, the AhR signaling pathway has been extensively studied in various fields, including biology, immunology, and pathophysiology (2). The AhR protein is able to interact with numerous endogenous and exogenous ligands. Exogenous ligands, such as 2, 3, 7, 8-tetrachlorodibenzo-p-dioxin (TCDD) and various viruses, can activate the AhR signaling pathway in host cells (3–5). Endogenous AhR ligands, such as Kynurenine (Kyn) and arachidonic acid, induce the translocation of AhR from the cytoplasmic matrix to the nucleus, thereby regulating the transcription of various target genes (6). Activation of AhR leads to the upregulation of cytochrome P450 enzymes, including CYP1A1 and CYP1B1, which play a crucial role in facilitating detoxification and drug metabolism (2). In the context of host-microbe interactions, AhR has the ability to collaborate with various signaling pathways, including signal transducer and activator of transcription 3 (STAT3), nuclear factor kappa B (NF-κβ), epidermal growth factor receptor (EGFR), and hypoxia-inducible factor-1α (HIF-1α) signaling pathways (7). In parallel with the significant advancement of the AhR signaling pathway, there has been a gradual improvement in several hypotheses concerning microbial infection. The role of AhR extends beyond its involvement in pathogen infection, encompassing immune system regulation such as the promotion of Treg cell generation (8), mediation of autophagy-related neurotoxicity (9), regulation of circadian rhythm, and initiation of carcinogenesis processes (7, 10). However, the precise mechanisms underlying these phenomena require further investigation in future research. Additionally, existing research indicates that the molecular chaperone heat-shock protein 90 (HSP90) plays a role in the activation of the AhR by forming a complex with co-chaperones HSP90, which then translocates to the nucleus (11). When AhR is inactive, HSP90, AhR, co-chaperone p23 (p23), and the hepatitis B virus X-associated protein (XAP2) bind together to form a stable cytoplasmic complex (12). This multiprotein complex is believed to enhance the structural stability of AhR and contribute to its subcellular localization.

Increasing evidence suggests that the AhR plays a crucial role in the invasion of microorganisms and the regulation of both innate and acquired immune responses to various microbial infections. AhR can regulate the expression of pro-inflammatory cytokines, including IFN-γ, TNF-α, and various interleukins. The enzyme indoleamine 2,3-dioxygenase-1 (IDO-1), involved in tryptophan metabolism, can be stimulated by inflammatory cytokines such as IFN-γ, leading to the production of kynurenine (Kyn) and promoting the antiviral activity of AhR (13, 14). A previous study conducted by Drozdzik et al. has demonstrated that IL-1 and TNF-α have the ability to regulate the functional expression of AhR in a human salivary cell line (15). Furthermore, AhR has been found to modulate the vasoactive apelin-APJ peptide system4, thereby providing prophylactic protection against dysplastic and apoptotic responses in respiratory epithelial cells, ultimately safeguarding the integrity of the lung barrier (16). The role of AhR in toxicology has been recognized throughout history, but it has been increasingly recognized as an important disease modulator in recent decades. AhR is reported to be associated with many diseases driven by immune and inflammatory processes. This review mainly discusses the role of AhR in inflammation and infection. We will also discuss the mechanisms of widespread involvement of AhR during infection of pathogens, including viruses, bacteria, fungi, and parasites, which can be retrieved until now.

Furthermore, the examination of the antagonist and agonist of AhR has brought about promising opportunities for development, leading to an accumulation of research in this area. Numerous prevalent nutraceuticals contain AhR inhibitor components, including folate, vitamin B12, and curcumin, which have the potential to regulate viral pathophysiology (17). The effective utilization of AhR antagonists could significantly address the issue of multi-drug resistance in viral infections in the foreseeable future. This review aims to summarize the significant role of AhR in invasive agent infections and its potential as a novel therapeutic target for microbial infections.

## The role of AhR in viral infection

### AhR and coronavirus infections

Coronaviruses (CoVs) are a group of positive sense single-stranded RNA viruses capable of infecting a wide range of hosts. Among them, Coronavirus 2 (SARS-CoV-2) is the causative agent of Corona Virus Disease (COVID) 19, a severe acute respiratory syndrome that emerged in China in 2019 (18). Inhibition of the AhR has been found to reduce the proliferation of both HCoV-229E (human Corona Virus 229E, a type of Corona Virus that is less pathogenic and usually causes respiratory symptoms) and SARS-CoV-2, suggesting that AhR activation serves as a mechanism for coronaviruses to evade the immune response and promote viral replication (3). The bioflavonoid dihydroxyflavone pinostrobin (PSB) functions as a negative modulator in the AhR/CYP1A1 signaling pathway by influencing the catabolic pathways of linoleic and arachidonic acid, thereby exhibiting antiviral effects (19). It has been observed that various coronaviruses can activate AhR signaling in hosts, leading to an increase in the expression of AhR-pathway genes in patients infected with SARS-CoV-122 (4). The activation of the AhR induced by coronaviruses leads to the up-regulation of downstream AhR elements, resulting in the manifestation of “Systemic AhR Activation Syndrome” (SAAS), which includes symptoms such as inflammation, fibrosis, thromboembolism, and potentially multiple organ dysfunction syndrome or mortality (20). While many mechanisms of coronavirus infection have been extensively studied, this discussion primarily focuses on the interplay between SARS-CoV-2 infection and the AhR signaling pathway. By characterizing the mechanisms underlying SARS-CoV-2 pathogenesis and the interaction between the host immune system and the pathogen, we can facilitate future advancements in therapeutic approaches.

The SARS-CoV2 virus gains entry into host cells by means of the interaction between spike glycoprotein and angiotensin-converting enzyme 2 (ACE2), resulting in various respiratory symptoms (21). The activation of AhR can facilitate the infection of SARS-CoV-2 by upregulating ACE2 expression, in conjunction with an increase in viral nuclear protein (NP) expression (22). The administration of 6-formylindolo (3,2-b) carbazole (FICZ) and omeprazole (OMP), which are agonists of AhR, reveals that AhR activation can decrease ACE2 expression, thereby inhibiting SARS-CoV-2 infection (23). According to research findings, pelargonidin, a natural flavonoid, is capable of reducing the expression of ACE2 in colon inflammation through an AhR-dependent mechanism. Through its binding to a fatty acid binding pocket in the receptor binding region of the SARS-CoV-2 spike protein, pelargonidin hinders the interaction between SARS-CoV-2 and ACE2, thereby inhibiting virus proliferation. These conclusions are based on molecular docking studies (24). Additionally, the study suggests that cigarette smoke condensates (CSC) may play a role in the regulation of ACE2 and transmembrane serine protease 2 (TMPRSS2) expression, potentially leading to SARS-CoV-2 infection by activating the AhR signaling pathway in gingival epithelial cells. It appears that CSC treatment may enhance the internalization of SARS-CoV-2 pseudovirus, as ACE2 is known to influence this process (25). In conclusion, these findings indicate that further investigation into the role of AhR is warranted in order to identify potential therapeutic strategies for managing SARS-CoV-2 infection.

**Figure 1:**
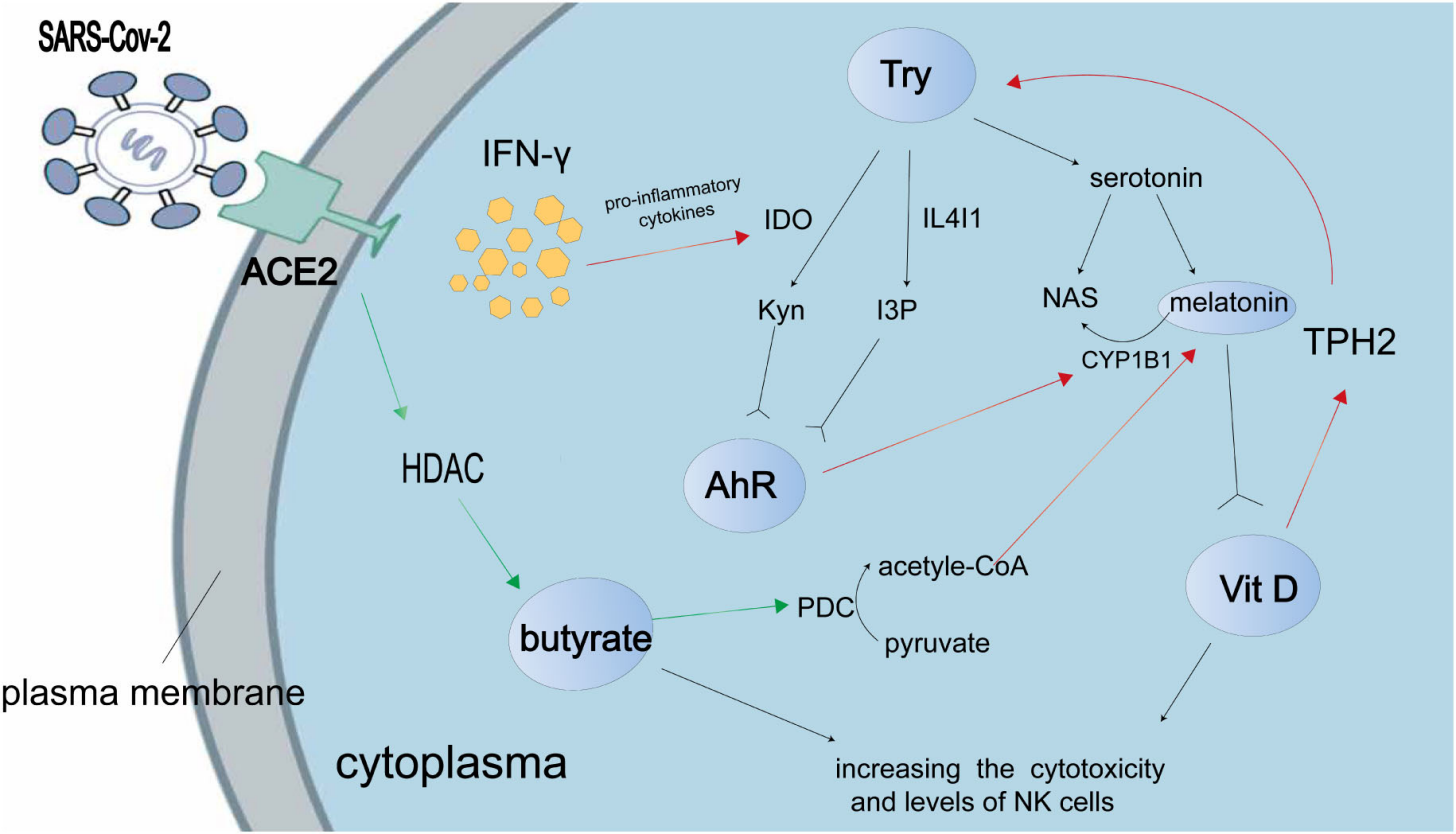
The present study shows the interaction between tryptophan metabolites and the AhR in the context of SARS-CoV-2 infection. The accompanying illustration illustrates the mechanism by which the SARS-CoV-2 virus modulates tryptophan metabolism to enhance the production of AhR ligands, namely kynurenine (Kyn), indole 3 pyruvate (I3P), and serotonin. Notably, Kyn and I3P serve as ligands for AhR. The SARS-CoV-2-induced “cytokine storm” leads to an upregulation of pro-inflammatory cytokines, which in turn promotes Kyn production through the enzyme indoleamine 2,3-dioxygenase (IDO). Consequently, this indirect effect results in a reduction in melatonin production. Activation of the AhR enhances the induction of cytochrome P450 1B1 (CYP1B1), which in turn facilitates the conversion of melatonin to N-acetylserotonin (NAS). However, during SARS-CoV-2 infection, the levels of butyrate are suppressed due to the inhibition of butyrate’s histone deacetylase (HDAC) activity. Butyrate exerts its effects by reducing the activity of the pyruvate dehydrogenase complex (PDC), leading to the conversion of pyruvate to acetyl-CoA, which serves as a co-factor in the melatonergic pathway. According to research findings, it has been observed that vitamin D has the ability to up-regulate the expression of tryptophan hydroxylase (TPH) 2, resulting in an increased production of tryptophan (Try) and subsequently leading to an elevation in serotonin synthesis. Additionally, melatonin has the capacity to bind to the vitamin D receptor and subsequently enhance the transcriptional activity mediated by vitamin D. It is important to note that the red arrow signifies a positive effect, whereas the green arrow represents a negative effect.

The disease known as Post-Acute Sequelae of SARS-CoV-2 infection (PAS) arises from the viral infection of SARS-CoV-2 and is characterized by persistent and severe acute respiratory symptoms lasting for a duration of three months or longer (26). Recent research findings suggest that the brain tissue of patients with PASC exhibits a significant expression of indoleamine 2, 3-dioxygenase-2 (IDO2), which plays a role in modulating SARS-CoV-2 infection through autophagy and apoptosis (27). This modulation could potentially be alleviated by the administration of an AhR antagonist. It is worth noting that the activation and expression of IDO-2 are triggered by the metabolites of tryptophan, specifically the Kynurenine generated by IDO-1. The Kynurenine/AhR/IDO-2 axis is implicated in the early initiation of severe SARS-CoV-2 viral infection, whereby the activation of IDO-2 and AHR leads to cellular responses such as apoptosis and autophagy in individuals with severe COVID-19 (28). Additionally, the SARS-CoV-2 viral infection has the ability to activate AhR through various mechanisms, including the induction of indole 3 pyruvate (I3P) by interleukin (IL) 4-inducible1 (IL41), resulting in a shift in the immunological mechanisms involved in SARS-CoV-2 infection (**Figure 1**). The increase in Kynurenine leads to a decrease in serotonin, which is synthesized from tryptophan and plays a crucial role in the melatonergic pathway in COVID-19. The main mechanism of SARS-CoV-2 infection involves the AhR-induced suppression of pineal melatonin, resulting in melatonin-insensitive modulation of mitochondrial metabolism. This suggests that melatonin and AhR antagonists could potentially serve as prophylactic therapies for severe SARS-CoV-2 infection. Additionally, vitamin D and butyrate also contribute to the disruption of gut microbiota caused by COVID-19, as they can increase the toxicity and number of NK cells (17).

There are several additional mechanisms mediated by AhR in the context of SARS-CoV-2 infection. Notably, one study shows that Fc signaling were enriched in AHR-dependent transcriptional modules, as revealed by pathway enrichment analysis of AhR-dependent and AhR-independent components in transcriptional responses to coronavirus infection. The AhR signaling pathway primarily inhibits the regenerative activity of lung epithelial basal cells, which play a crucial role in tissue repair (29). This inhibition leads to the accumulation of stem cells and facilitates the establishment of coronavirus infection. Coronaviruses have been observed to induce the activation of AhR through a pathway that is not dependent on IDO1. This activation leads to an increase in the expression of TCDD-inducible poly (ADP-ribose) polymerase (TiPARP), which serves as a downstream effector and modulates the expression of cytokine genes (4). Additionally, SARS-CoV-2 infection has been found to stimulate the AhR signaling pathway, thereby promoting viral replication by upregulating the transcription of ACE2 and dampening the antiviral immune response mediated by IFN-I. The activation of AhR in the IFN/IDO/Kyn axis by the viral infection leads to lung inflammation, which subsequently promotes the expression of mucin production, intercellular adhesion, and cytokine release. Ultimately, this process contributes to the increased replication of SARS-CoV-2 (30). Consequently, AhR emerges as a significant host factor for SARS-CoV-2-infected cells and a potential target for antiviral therapies against SARS-CoV-2. Therefore, it is crucial to further investigate and comprehend the underlying mechanisms that govern SARS-CoV-2 infection for future research endeavors.

### The role of AhR in EB virus infection

The Epstein-Barr virus (EBV), a prevalent type of herpes virus, is capable of persisting in memory B cells without causing symptoms (31). However, there is still a potential risk for the development of autoimmune diseases, such as sicca syndrome (SS), due to EBV-associated cellular transformation and subsequent viral invasion. The activation of the EBV infection from a latent to a lytic state is facilitated by TCDD, which induces the transcription of BZLF1. The reactivation of EBV, triggered by AhR ligands, plays a role in the development of SS in EBV-positive salivary epithelial cells and B cells. Inoue et al. postulate that the induction of EBV reactivation by TCDD may enhance the immune response in the salivary glands of patients with SS (32). This is achieved through the involvement of latent membrane protein 1 (LMP1), which binds to TRAF1-3 and facilitates the interaction between the NF-κB pathway and AhR signaling pathway during the B-cell transformation process initiated by EBV invasion (33). Further investigations have demonstrated that EBV possesses the capability to induce human lymphomas and various carcinomas, including gastric cancer (GC) (34). In EBV-positive gastric carcinoma (GC) cells, it has been observed that latent membrane protein 2A (LMP2A) activates the phosphorylation of extracellular signal-regulated kinase (ERK), thereby inhibiting the expression of AhR through the MAPK/ERK pathway. LMP2A is considered one of the most crucial molecules involved in the carcinogenic transformation of EBV, as it plays a role in maintaining the viral latent state and modulating intracellular signaling pathways. The downregulation of AhR expression can be observed in EBV-positive GC tissues, thereby attenuating the pro-proliferative effect and nuclear translocation (35). The examination of the interactions between the Epstein-Barr virus (EBV) and the AhR signaling pathway warrants comprehensive exploration, as it holds significant potential for advancing therapeutic approaches in the treatment of EBV-associated cancers.

### AhR and influenza A virus infection

The influenza virus, particularly influenza A virus (IAV), is a pathogen that frequently leads to global pandemics and mortality. Numerous epidemiological studies have demonstrated a correlation between AhR activation and the severity of respiratory infections (36). The activation of AhR has the potential to influence influenza virus infection through the responses of various lymphocytes, including CD4^+^ T cells, dendritic cells, T follicular helper cells, and CD8^+^ T cells. The forthcoming discussion will provide a detailed description of the interactions between these lymphocytes and AhR. The persistence of CD4^+^ T cell responses to influenza virus infection is attributed to the binding of AhR ligands, which directly affect CD4^+^ T cells (37). Research has demonstrated that AhR not only influences CD4^+^ T cells during infection but also impacts the patterns of DNA methylation in these cells (38). Analysis of gene and protein expression reveals that the expansion and function of CD4^+^ T cells are suppressed during viral infection due to increased AhR activation. Furthermore, the treatment of mice with an AhR agonist during IAV infection results in differential methylation of seven out of eleven genes in CD4^+^ T cells. Following treatment with drugs that alter DNA methylation, various functions of CD4^+^ T cell responsiveness were restored. These findings suggest that DNA methylation may serve as a mechanism regulated by AhR, capable of consistently modifying T cell functions in mice (38). Previous research has elucidated that a similar mechanism can be observed in CD8^+^ T cells, where increased AhR activation leads to genomic variations in DNA methylation patterns (39). Notably, developmental AhR activation visibly diminishes the polyfunctionality of cytotoxic T lymphocytes (CTL) and alters the transcriptional progression of CD8^+^ T cells (40). S-adenosylmethionine (SAM) has been found to enhance polyfunctionality and augment the population of specific virus CD8^+^ T cells by promoting DNA methylation. It is postulated that the reduction in methylation induced by AhR-binding ligands may lead to persistent alterations in antiviral CD8^+^ CTL functions (40). In the context of influenza A virus (IAV) infection, AhR negatively modulates host immune responses by inhibiting dendritic cell (DC) activity in priming naive CD8^+^ T cells, thereby diminishing the generation of CD8^+^ CTL (41). Moreover, the AhR presents a promising avenue for manipulating T follicular helper (Tfh) cell reactions, thereby reducing the development of Tfh cells and T cell-dependent B cell responses during influenza virus infection (42).

Furthermore, the activation of the AhR not only affects lymphocytes but also leads to neutrophilia in peripheral tissues (43). Among the various agonists for AhR, the 2, 3, 7, 8-tetrachlorodibenzo-p-dioxin (TCDD) is considered one of the most potent, exacerbating inflammatory pulmonary diseases upon exposure to AhR ligands. Different AhR ligands, such as TCDD and 6-formylindolo (3, 2-b) carbazole (FICZ), can induce immunomodulatory variations in response to influenza A virus infection, depending on the duration of AhR activation and the specific types of activated cell receptors (44). The activation of the AhR by 2,3,7,8-tetrachlorodibenzo-p-dioxin (TCDD) results in an increased number of neutrophils in the lung during infection with influenza A virus (IAV) (45). Recent studies indicate that extrinsic bone marrow-derived cells, mediated by AhR, play a role in regulating the directed migration of neutrophils, which specifically increase at the site of antigen challenge in response to pulmonary infection (46). Additionally, our findings demonstrate that AhR activation enhances the accumulation of neutrophils in the lung and upregulates the expression of inducible nitric oxide synthase (iNOS) within the same context (47). The nuclear translocation of AhR is dependent on its binding to the intrinsic DNA domain, facilitating infection-associated accession in iNOS expression and neutrophil recruitment. Notably, the increase in these two factors is not correlated, indicating that AhR activation yields independent outcomes (48). These findings demonstrate the role of AhR in regulating the equilibrium between moderate and excessive inflammation in the context of influenza A virus infection.

**Figure 2:**
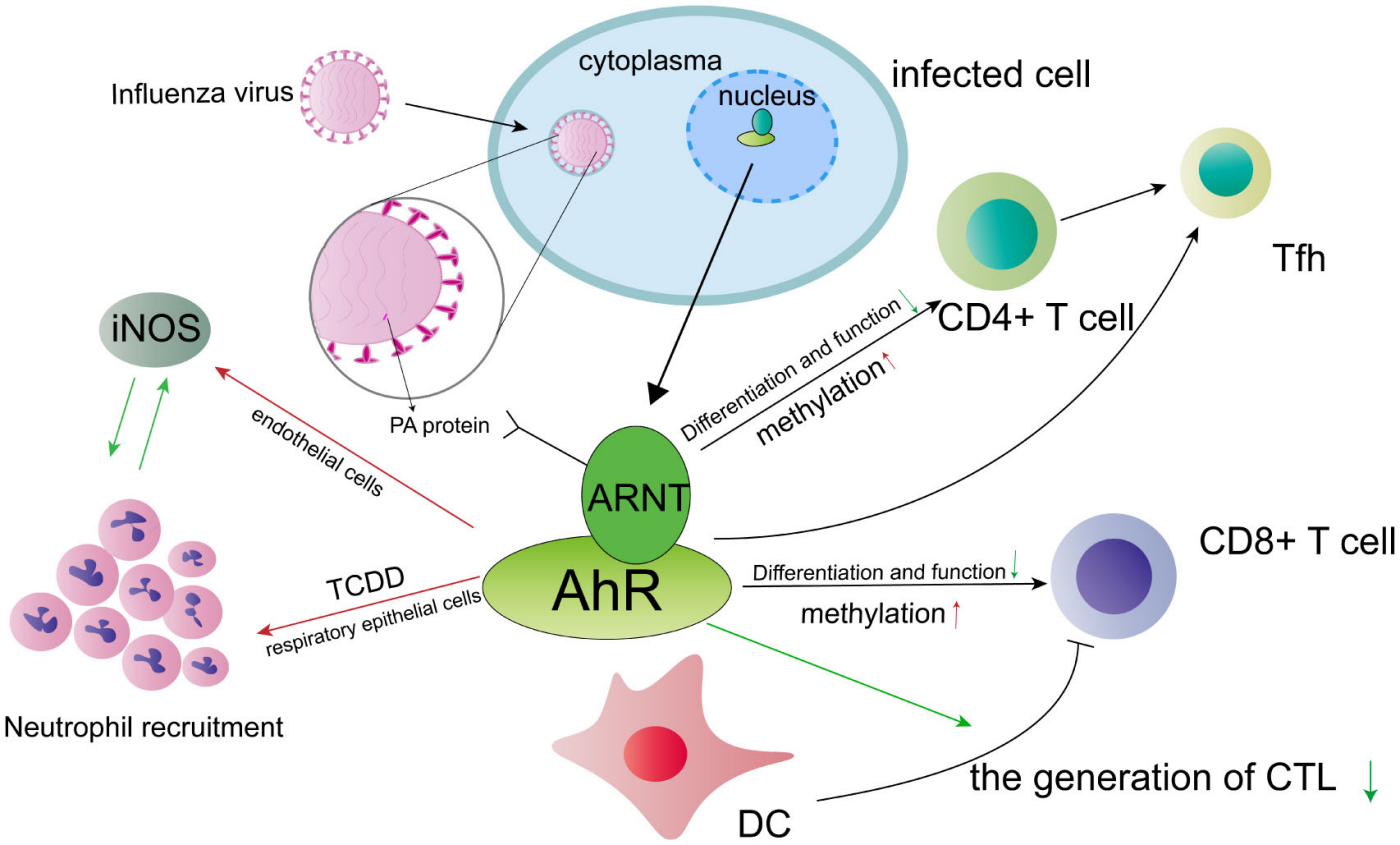
The involvement of the AhR in influenza A virus (IAV) infection is of significant interest. Activation of AhR has been shown to influence the response of various cell types, potentially impacting the course of IAV infection. Notably, AhR not only affects the function and differentiation of CD4^+^ T cells during IAV infection, but also influences DNA methylation patterns in these cells. Similar mechanisms may be observed in CD8^+^ T cells. Furthermore, AhR presents a promising target for modulating T follicular helper (Tfh) cell responses, offering a potential avenue for therapeutic intervention. During infection with IVA, the AhR exerts a suppressive effect on the host’s immune responses by negatively regulating the function of dendritic cells (DCs) in their ability to prime naive CD8^+^ T cells. Consequently, this may lead to a reduction in the number of cytotoxic T lymphocytes (CTLs). Additionally, the AhR agonist, TCDD, enhances the expression of inducible nitric oxide synthase (iNOS) and promotes the recruitment of neutrophils in endothelial and respiratory epithelial cells, respectively. However, it is important to note that there exists a reciprocal inhibition between iNOS and neutrophils. ARNT interacts with the polymerase acidic (PA) protein, thereby exerting a significant influence on the viral pathogenicity of the influenza virus. The red arrow symbolizes a positive effect, whereas the green arrow signifies a negative effect.

The Aryl hydrocarbon receptor nuclear translocator (ARNT) is known to bind to AhR and facilitate its nuclear transportation (49). Recent studies have indicated interaction between ARNT and the polymerase acidic (PA) protein of the H5N1 virus (50). The PA subunit is a complex protein that plays a crucial role in viral pathogenicity by regulating the replication and transcription of the IAV (51). Through a yeast two-hybrid screen, ARNT has been identified as a novel host factor that interacts with the PA protein. Specifically, the bHLH/PAS region of ARNT primarily interacts with the C-terminal domain of the PA subunit. During this interaction, the overexpression of ARNT was found to have a significant impact on the downregulation of polymerase activity, thereby limiting the propagation and transcription of the influenza viral genome. This ultimately led to the accumulation of the PA protein in the nucleus (50). In conclusion, the activation of AhR has the potential to influence influenza virus infection through various mechanisms (**Figure 2**). AhR plays a regulatory role in the functions and responses of CD4^+^ T cells, dendritic cells, CD8^+^ T cells, and neutrophils during influenza virus infection. ARNT binds to AhR and interacts with the C-terminal domain in the PA subunit of the H5N1 virus, ultimately resulting in the nuclear accumulation of the PA protein. In future studies, we intend to investigate additional pathways that are specific to these cell lineages and their interaction with AhR-dependent regulatory signals.

### The role of AhR in respiratory syncytial virus infection

Respiratory syncytial virus (RSV) is a prominent causative agent of lower respiratory tract infection, with the potential to precipitate early and recurrent pulmonary disease. Prior research has demonstrated that the AhR plays a role in modulating immune responses to respiratory viral infections by regulating the interplay among different cell types, such as dendritic cells (DCs) (52). *In vivo*, DCs serve as direct recipients of AhR ligands, and their accumulation is associated with heightened allergen sensitization and exacerbated pathology in the context of RSV infection. The impacts of respiratory syncytial virus (RSV) infection encompass the monitoring of pathophysiological AhR activity, resulting in excessive mucus production and heightened Th2 cytokine responses (53). Additionally, there exists an interplay between bacteria and viruses, which synergistically contributes to the development of severe disease and clinical symptoms (54). Lipopolysaccharide (LPS), acting as the ligand for Toll-like receptor 4 (TLR4), interacts with RSV infection to amplify inflammatory reactions, modulate AhR signaling, and alter cytokine profiles. TLR4 activation can be induced by LPS, leading to enhanced viral clearance. Consequently, the level of TLR4 activation can serve as an indicator of disease severity in RSV infection. The interaction between RSV and LPS triggers AhR signaling and respiratory tract inflammation through the TRIF-MMP-9-neutrophil-MMP-9 signaling pathway (55). Consequently, inhibiting this co-infection interaction may prove beneficial in the development of vaccines and therapies for RSV infection in the future.

### The role of AhR in hepatitis C virus infection

The hepatitis C virus (HCV) pathogen is known to cause hepatitis C and contribute to the development of hepatocellular carcinoma and other severe liver diseases. Recent analysis suggests that HCV-induced hepatocellular carcinoma tumors exhibit an increased expression of genes involved in the AhR signaling pathway (56). Consequently, AhR emerges as a pivotal factor in HCV infection.

Hepatitis C virus (HCV) exerts control over the metabolic processes of host cells, leading to the formation of specific cytomembrane structures, namely lipid droplets (LDs). This manipulation facilitates the replication of the virus and the efficient assembly of viral particles (57). The AhR serves as a target for flutamide and plays a pivotal role in regulating the accumulation of LDs and the generation of HCV in hepatic cells (58). Additionally, the cytochrome P450 (CYP) family of genes, downstream targets of AhR, actively participate in the metabolism of xenobiotics, with CYP1A1 being a notable example (59). The findings of the study suggest that inhibiting the AhR-induced CYP1A1 enzyme can effectively reduce the excessive production of lipid droplets. Conversely, increasing the levels of CYP1A1 in AhR-inactivated cells can restore lipid and triglyceride accumulation. Additionally, the antagonist Flutamide, which targets the AhR, impairs the host cell’s capacity to generate HCV by disrupting lipid accumulation. In conclusion, the findings of Ohashi et al. provide evidence that HCV promotes the activation of the AhR/CYP1A1 pathway, leading to the reorganization of hepatic cell functions and subsequent accumulation of lipid droplets (58). This process ultimately plays a crucial role in HCV assembly. Therefore, targeting this pathway could serve as an effective strategy for combating HCV infection and hepatic LDs overproduction.

The AhR transcription factor is highly conserved and has been found to play a significant role in promoting tumorigenesis and carcinogenicity in transformed host cells. This occurs through the suppression of apoptosis and the disregarding of AhR-dependent cell growth arrest when activated by its endogenous ligand, Kynurenine (60). Tian et al. have provided a description of AhR’s ability to modulate the toxicities of dioxins and similar compounds (61). Additionally, Opitz et al. have demonstrated that the endogenous ligand of AhR, derived from TDO, Kyn, can down-regulate the anti-tumor effects and enhance the survival rate of tumor cells through the AhR pathway (62). It is hypothesized that HCV infection may expedite the development of hepatocellular carcinoma (HCC) by amplifying the tryptophan (Trp)-TDO-Kyn-AhR pathway, thereby impeding immune responses against viral and tumor antigens, ultimately leading to tumorigenesis (63). The persistence of HCV infection induces chronic inflammation, attracting dendritic cells and macrophages, which in turn elevate the levels of IDO in the liver. Consequently, the upregulation of IDO contributes to the metabolic conversion of Trp into Kyn through TDD, facilitating the activation of AhR. The activation of AhR signaling, in conjunction with cytokine signaling, induces the differentiation of naive CD4^+^ T cells into Tregs. Tregs possess the capability to suppress immune responses, thereby preventing the formation of hepatocellular carcinoma (HCC) (63). During the progression of HCC establishment, it has been observed that AhR signaling is heightened in hepatocytes during HCV infection. However, the precise mechanisms governing pathogen immune evasion through the TDO-Kyn-AhR pathway require further elucidation.

A previous study has indicated that MicroRNA (MiRNA) plays a significant role in HCV proliferation and lipid metabolism, thereby limiting the progression of liver fibrosis and exhibiting potential antitumor effects (64). Additionally, the expression of miR-10a follows a circadian pattern and influences the expression of the brain and muscle aryl hydrocarbon receptor nuclear translocator-like 1 (Bmal1), which is involved in circadian gene regulation (65). This modulation occurs through the downregulation of the RA receptor-related gene, which serves as a target gene for miR-10a. The interaction between miR-10a and Bmal1 is crucial for the regulation of circadian rhythm. MiR-10a plays a significant role in the catabolism of lipids, protein, glucose, and bile acid through the down-regulation of Bmal1 genes. It also regulates fatty acid metabolism by inhibiting the expression of sterol regulatory element binding protein (SREBP1), SREBP2, and fatty acid synthase (FASN), as well as other lipid synthesis genes. Additionally, MiR-10a modulates gluconeogenesis through peroxisome proliferator-activated receptor gamma coactivator 1 alpha (PGC1α), protein synthesis through mammalian target of rapamycin (mTOR) and ribosomal protein S6 kinase (S6K), and bile acid synthesis through liver receptor homolog 1 (LRH1). The downstream genes of Bmal1, which are regulated by miR-10a, exhibit a strong association with the exacerbation of pathological liver catabolism in individuals with chronic hepatitis C (65). Importantly, miR-10a holds promise as a potential effective biomarker for assessing the prognosis of liver cirrhosis in forthcoming studies.

### The role of AhR in human immunodeficiency virus infection

Human immunodeficiency virus (HIV), the causative agent of acquired immunodeficiency syndrome (AIDS), disrupts the equilibrium of the immune system and facilitates the occurrence of opportunistic infections. HIV induces chronic inflammation and immune dysfunction, resulting in diverse modifications to cellular catabolic pathways, including glucose, lipids, and amino acids (66). To impede the advancement of HIV-1 infection, the regulation of cellular metabolic pathways presents a viable approach. Studies indicate that the Kynurenine tryptophan metabolite, acting as an endogenous ligand of AhR, contributes to the accelerated progression of HIV-1. AhR facilitates HIV-1 transcription by binding to the 5ˊ-long terminal repeat domain of the virus and amplifies the accumulation of the positive transcription elongation factor complex, thereby phosphorylating RNA Pol II (67). It is postulated that targeting the downstream effects of AhR may be pivotal in controlling HIV-1 infection. Conversely, AhR can also serve as a hindrance to HIV-1 infection by means of CD4^+^ T cells. The observed reduction in IL-22, IL-17A, and IL-10 production in AhR knockout cells suggests that AhR actively facilitates HIV-1 replication. This analogous mechanism is observed in the pharmacological group of AhR, wherein antagonists are employed to impede the binding of AhR and its ligands. The subsequent cohort additionally impedes the replication of HIV-1 during the stages of integration and reverse transcription within memory CCR6^+^ CD4^+^ T cells. Furthermore, we have identified through chromatin immunoprecipitation that HIV-1 is a target of AhR. These findings manifest as the facilitation of HIV-1 replication in CD4^+^ T cells of individuals living with HIV-1 who are undergoing antiretroviral therapy (ART). Consequently, AhR exerts control over T cell transcription *via* tissue circulation and outgrowth, thereby representing a significant therapeutic approach in the management of individuals living with HIV-1 (68).

### The role of AhR ligand in human cytomegalovirus infection

Human cytomegalovirus (HCMV), a member of the herpesvirus family, modulates the activation of cellular pathways involved in metabolism in order to facilitate its own replication. Specifically, HCMV enhances glycolysis and lipid synthesis in target cells (69). Notably, Kyn levels, an endogenous ligand of AhR, are significantly increased in fibroblasts infected with HCMV (70). The presence of unidentified metabolites such as Kyn may potentially impact HCMV replication and provide novel insights into the intricate interplay between HCMV and host cell metabolism. The reduction of AhR has the potential to decrease the replication of HCMV, whereas the activation of AhR using an exogenous ligand can enhance virus proliferation. Wise et al. propose that Hypoxia-inducible factor 1α plays a significant role in the antiviral response by inhibiting the synthesis of Kyn and activating AhR during HCMV infection (71). Additionally, HCMV infection regulates the activation of AhR, which in turn modulates the transcription of infected cells. In a precise manner, it can be stated that the AhR plays a crucial role in facilitating the HCMV-induced G1/S block, which effectively hinders cellular DNA replication and consequently impedes the progression of infected fibroblasts into the S phase. By employing two distinct locked nucleic acids to suppress AhR expression, it becomes evident that the accumulation of viral DNA is significantly reduced. Hence, it can be inferred that the activation of AhR by Kyn during HCMV infection serves as a mediator for essential functions in the interaction between the virus and the host (72). These investigations present a unique perspective on a previously unexplored aspect of the mechanism underlying the interaction between the virus and the host cell over an extended period of time.

### AhR and herpesvirus infection

Herpes simplex virus 1 (HSV-1) is a pathogen capable of infecting epithelial cells and invading neurons (73). The reactivation of latent herpesvirus infections is commonly observed in individuals with compromised immune systems. Recent research indicates that the AhR signaling pathway plays a role in suppressing the production of IL-17 in mice infected with gamma-herpesvirus 68, resulting in a weakened immune response (74). The levels of indolamine 2-3 dioxygenase (IDO1) and tryptophan dioxygenase (TDO2), which are key components of the Kyn pathway and AhR ligands, are essential for the replication of herpesvirus in fibroblasts. Lung dendritic cells (DCs) expressing AhR and CD103 are observed to accumulate in response to TDO2-expressing fibroblasts, replacing AhR^+^ lymphocytes. This suggests that the fibroblast (TDO2)/DC (AHR) axis plays a crucial role in herpesvirus infection (74). Additionally, we have observed an elevated level of interferon (IFN) signaling upon genetic ablation of AhR during HSV-1 infection. HSV-1 has the ability to exploit host mechanisms to evade antiviral defenses and enhance its own replication. The impact of AhR agonist treatment on interferon regulatory factor 3 (IRF-3) rather than IFN-β is responsible for the observed enhanced resistance to HSV-1 infection in treated cells (75). The specific mechanism by which IRF-3 influences HSV-1 infection is currently unknown, but studies indicate a similar mechanism exists in human cytomegalovirus (76). Consequently, the use of AhR antagonists at the site of invasive infection may serve as a potential therapeutic approach for combating viral infections, as AhR plays a negative role in restricting cell-autonomous antiviral resistance.

### The role of AhR in other viral infections

In addition to the extensive survey conducted, numerous other viral infections are linked to the AhR signaling pathway, including BK polyomavirus, Zika virus, Junín virus, and others. BK polyomavirus infection is frequently observed following kidney transplantation, leading to the development of BK polyoma virus allograft nephropathy (BKPyVN) (77). Given AhR’s role in drug metabolism, this transcription factor regulates the polarization of macrophages by influencing viral tolerance and inflammatory response. Based on the findings of the study on renal immune response, it has been observed that the expression of AhR in the BKPyVN group is significantly elevated compared to the control group with T-cell mediated rejection (TCMR). Furthermore, analysis of the infiltrating graft biopsies reveals an increased presence of AhR in the nuclei of CD45^+^ and CD68^+^ cells in BKPyVN cases. These findings suggest a potential interplay between adaptive and innate immunity, which may contribute to a deeper comprehension of BKPyVN (78). Zika virus (ZIKV) infection is associated with mild symptoms but can result in various birth defects, notably microcephaly, which has garnered significant attention regarding the treatment of ZIKV (79). Recent research indicates that ZIKV infection induces the activation of the aryl hydrocarbon receptor (AhR) through the production of Kyn, as observed in genome-wide transcriptional studies. This activated AhR subsequently hampers ZIKV replication by constraining the intrinsic immune response mediated by the promyelocytic leukemia (PML) protein. Persistent study of AhR inhibition is warranted due to its potential to reduce the replication of both ZIKV strains *in vitro* and other related flavivirus dengue (29). Additionally, AhR plays crucial roles in Human T-cell leukemia virus type 1 (HTLV-1) plus-strand transcription by directly binding to the HTLV-1 LTR dioxin response element (DRE) site (CACGCATAT). Furthermore, the manipulation of AhR overexpression in HTLV-1 infected T-cells through the activation of the nuclear factor kappa B (NF-κB) pathway, as well as the level of AhR ligands Kyn, determines HTLV-1 latency-reactivation-latency (80). The adenovirus-dioxin-responsive bioassay system proves to be a valuable tool for the detection of dioxins, as it relies on the AhR responsiveness of adenovirus (81). Additionally, it has been observed that the AhR signaling pathway can exert pharmacological control over the replication of Junín virus (JUNV). In JUNV-infected hepatic cells, an upregulation of AhR has been identified, and through immunofluorescence detection, it has been demonstrated that the replication of JUNV strains can be dose-dependently inhibited by an AhR antagonist (82). Future research could focus on the exploration of unidentified modulating AhR ligands as a means of mitigating harmful immune responses.

## The role of AhR in bacterial infection

**Figure 3:**
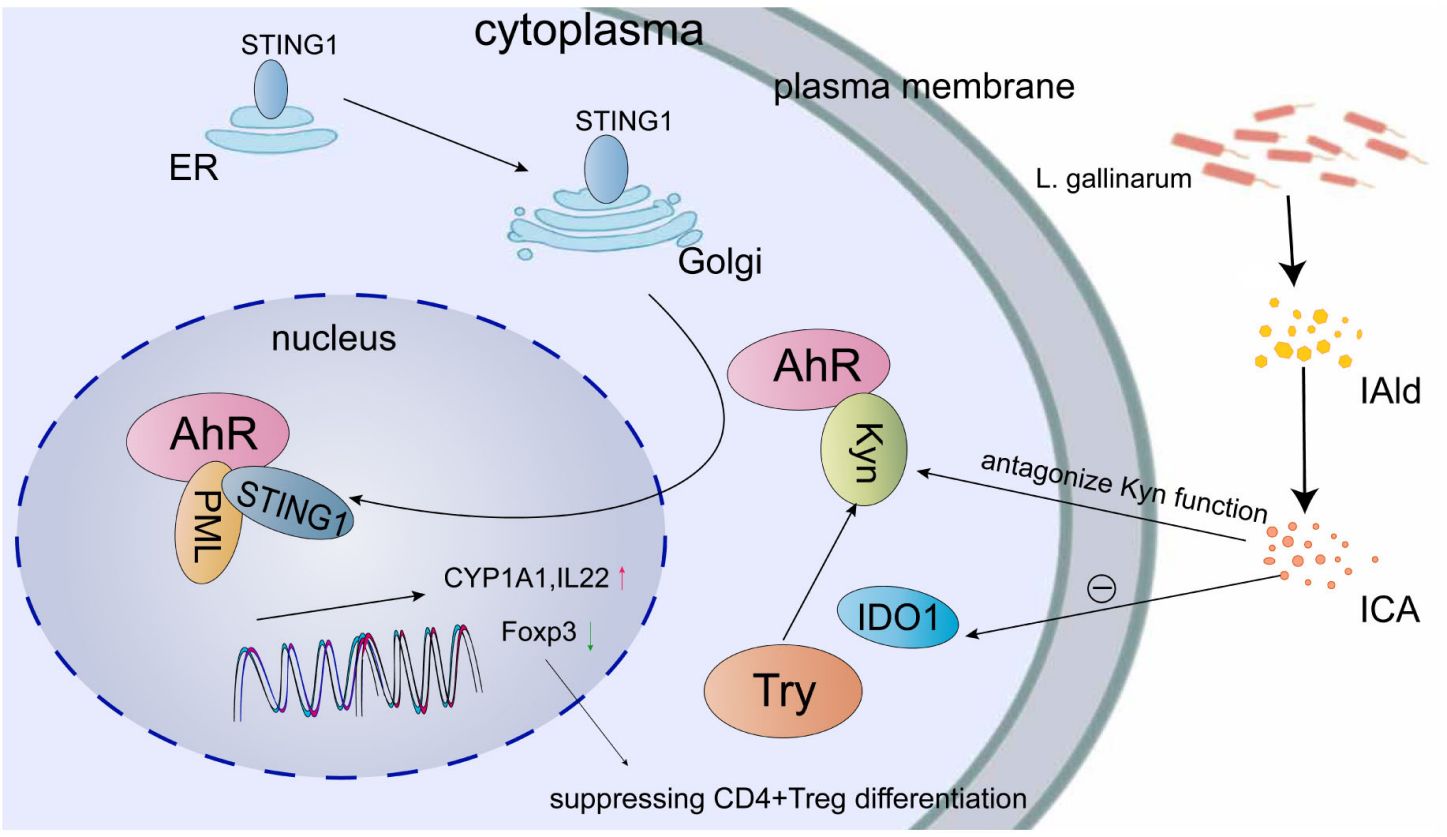
Multiple mechanisms are involved in the regulation of AhR for maintaining gut microbiota homeostasis. One such mechanism involves the translocation of STING1 from the endoplasmic reticulum (ER) to the Golgi apparatus, followed by its subsequent movement into the nucleus through AhR and AhR ligands. The formation of the STING1-AhR complex requires the presence of positive regulators such as PML, which facilitate the expression of CYP1A1 and IL22. Indole-3-carboxaldehyde (IAld) and ICA, which are synthesized by *L. gallinarum*, have been found to suppress the expression of IDO1 and counteract the function of Kyn, consequently leading to a reduction in Foxp3^+^ transcription. This reduction in transcription may potentially hinder the differentiation of CD4^+^ Treg cells.

Increasing evidence suggests that AhR plays a significant role in the pathogenesis of various bacteria, contributing to the development of inflammatory bowel disease. There is a growing recognition that AhR can enhance the integrity of the intestinal immune and physical barriers by regulating the expression of tight junction proteins and reducing cecal inflammation (83). Moreover, AhR activation exerts a multifaceted influence on gut microbiota homeostasis through intricate mechanisms (**Figure 3**). The Stimulator of interferon genes (STING1) is a protein that interacts with guanosine mono-phosphate-adenosine monophosphate (cGAMP) and specifically interacts with the N-terminal region of AhR. When located in the nucleus, STING1 modulates AhR activation. The formation of the STING1-AhR complex requires the presence of nuclear partners such as RNF20 (ring finger protein 20), XRCC6 (X-ray repair cross complementing 6), DHX9 (DExH-box helicase 9), and PML (the only positive regulators). STING1 activation triggers the activation of AhR in the nucleus, leading to the improvement of intestinal homeostasis and the promotion of the expression of CYP1A1, IL22, and other related factors (84). Indole-3-carboxylic acid (ICA), a catabolic derivative of *Lactobacillus gallinarum*, competes with Kyn for binding sites on AhR and also suppresses IDO1 expression. The antagonistic effect of ICA on Kyn function results in the down-regulation of Foxp3^+^ transcription, indicating that ICA has the potential to regulate the IDO1/Kyn/AhR axis, thereby reducing the differentiation of CD4^+^Treg cells and enhancing the function of CD8^+^T cells (85).

The modification of the extracellular matrix, regulated in part by AhR-associated genes, may have notable implications for the integrity of the intestinal cell barrier and the immune response during *salmonella* infection in the host (86). Comparable functions can also be observed in distant organs, as AhR agonists derived from the photooxidation of Trp possess the capacity to ameliorate *Eschrichia coli*-induced endometritis by restoring functional barriers and mitigating inflammatory responses (87). The activation of AhR through *Lactobacillus reuteri* tryptophan metabolism plays a role in mitigating *Escherichia coli-*induced mastitis and maintaining host homeostasis by limiting NF-κB activation and improving barrier function (88). Additionally, *Streptococcus gallolyticus* interacts with AhR-mediated pathways involved in cellular cytochrome P450 (CYP) 1 biotransformation, potentially leading to DNA damage and effectively eliminating intestinal pathogens, thus protecting the epithelial barrier (89). The AhR molecule is involved in the cooperative influence of VD3 and butyrate on the integrity of the intestinal barrier against *Salmonella* infection. This is achieved by augmenting antibacterial responses and reducing the expression of tight junction proteins, thereby impeding pathogen invasion (90). However, it is important to note that AhR activation can have both beneficial and detrimental effects. Specifically, the activation of AhR by Benzo(a)pyrene has the potential to mitigate or eradicate septic peritonitis resulting from systemic *Salmonella enterica* infection (91). Furthermore, Zhu et al. have reported that *Helicobacter pylori* (*H. pylori*) infection hinders the expression of both AhR and aryl-hydrocarbon receptor repressor (AHRR) in the mucosa of the digestive tract, potentially leading to the progression of chronic inflammation and prolonged bacterial sustenance (92). In light of this, Soyocak et al. have postulated that AhR and AHRR may play a role in the gastric pathogenesis associated with *H. pylori* (93). Subsequently, they have discovered an increased expression of AhR in *H. pylori*-positive patients with chronic gastritis. The infection of macrophages by *Mycobacterium tuberculosis* triggers AhR signaling through the regulation of IL23A transcription, offering potential avenues for the treatment of pulmonary disease due to the high expression of AhR in the lungs (94). In terms of cutaneous defense, both *Staphylococcus epidermidis* and *Staphylococcus aureus* possess the capability to activate AhR in Human Keratinocytes, thereby inducing the gene expression of CYP1A1 and CY-P1B1 for cutaneous innate defense (95, 96). When the AhR signaling pathway is activated, *S. epidermidis* exhibits contrasting effects on skin cells derived from atopic and healthy skin (97). Additionally, AhR serves as a component of the host defense response in both *Pseudomonas aeruginosa* and *Pseudomonas plecoglossicida*, by binding to pyocyanin produced by *P. aeruginosa* and up-regulating the expression levels of cyp1a, respectively (98, 99). *Porphyromonas gingivalis* possesses the capacity to induce inflammatory responses through the inhibition of the AhR signaling pathway in periodontitis (100). The AhR pathway plays a role in maintaining cellular homeostasis by regulating tubulin dynamics. The obstruction of AhR by an excess of Trp-derived indole leads to a reduction in the expression of detyrosinated tubulin, thereby facilitating the growth of *Chlamydia trachomatis* (101). Collectively, these findings collectively offer substantiation for innovative therapeutic approaches concerning bacterial infection. However, a comprehensive investigation into the involvement of AhR in the pathogenic mechanisms of bacteria remains unpublished. Consequently, there is still a considerable distance to traverse in order to advance the intricacies of research efforts focused on AhR-targeted therapeutic interventions for inflammatory diseases.

## The role of AhR in parasites infection

Previous research indicates that AhR may limit parasite replication and infection outcome by functioning as a ligand-activated transcription factor in various regulatory pathways (102). However, the specific mechanisms through which AhR exerts its antiparasitic effect remain unclear. *Plasmodium falciparum*, the causative agent of malaria, is responsible for significant morbidity and mortality rates, particularly among the South African population. AhR signaling plays a critical role in malaria by maintaining the equilibrium between infection cell proliferation, immune response, and inflammation (103). Acute kidney injury (AKI) is a significant complication of malaria that is linked to heme metabolism and the generation of AhR agonists. The potential therapeutic approach of targeting AhR and heme metabolism shows promise in managing AKI by controlling parasitemia and mitigating tissue damage (104). Furthermore, based on research investigating the pathological progression of *P. berghei Anka*, it appears that AhR may also play a role in regulating parasite replication and inflammation (105). Infections caused by *Leishmania major* exhibit a higher prevalence and mortality rate among human infections, as indicated by the Neglected Tropical Diseases listed by the World Health Organization (106). The absence of AhR in mice infected with *L. major* resulted in reduced levels of Treg cells, elevated levels of Interferon (IFN)-γ and IL-12, and a decline in the secretion of IL-10 and IL-4 (107). Additionally, Münck et al. conducted a study that examined genome variations in granuloma macrophages, providing further evidence of the association between the AhR signaling pathway and early skin micromilieu in *L. major* infection. Furthermore, it has been demonstrated that treatment with AhR antagonists resulted in a decrease in tumor necrosis factor (TNF)-α mRNA expression induced by *L. major* in monocytes (108). These aforementioned studies have provided evidence of the diverse roles played by AhR in various parasites. Additionally, research has indicated that AhR is implicated in the inflammatory response to *Trypanosoma cruzi*, *Toxoplasma gondii*, *schistosomiasis japonica*, and *coccidium* through various mechanisms (102, 107, 109–111). After acquiring comprehension of the regulatory function of AhR in inflammation, it aids in the advancement of antiparasitic medications.

## The role of AhR in fungus infection

The precise mechanisms by which the AhR signaling pathway regulates the pathogenicity of fungal infections remain poorly understood. Paracoccidioidomycosis (PCM), caused by *Paracoccidioides brasiliensis*, is recognized as the most prevalent mycosis in Latin America (112). The immunological equilibrium mechanism, modulated by the IDO/AhR axis, plays a crucial role in maintaining the balance between Th17/Treg cells and the severity of lung PCM (113). The lung, acting as a barrier organ, expresses high levels of AhR. Research indicates that the involvement of IDO and AhR is essential for the control of pulmonary fungal infection and the tolerance of mycosis (114). The aryl hydrocarbon receptor (AhR) plays a crucial role in regulating the delicate equilibrium between the fungal-host relationship, commonly referred to as “commensalism vs infection” (115). Through its involvement in tryptophan metabolism in both the host and microbial communities, AhR effectively coordinates the activities of T cells (115). In this context, indoleamine 2,3-dioxygenase 1 (IDO1) assumes a significant role in orchestrating T-cell immune homeostasis at mucosal surfaces, which is contingent upon AhR. AhR and IDO1 may contribute to the development of a novel therapeutic approach that supports the survival of the host and the establishment of fungal commensalism. This is achieved by facilitating the coevolution of commensal fungal groups with human immunity and the microbiota (115). Various studies have suggested that AhR could potentially mediate the toxicity of ochratoxin A and aflatoxin B1. This is accomplished by promoting the expression of P450 and inducing oxidative stress in kidney cells, respectively (116, 117). Solis et al. reported that the AhR functions through Src family kinases to phosphorylate the epidermal growth factor receptor, thereby inducing the endocytosis of *Candida albicans* (118). Subsequent investigations have demonstrated that AhR can also be activated by Kynurenic acid, leading to the inhibition of the myosin light chain kinase-phospho-myosin light chain signaling pathway, which alleviates inflammation in the lower digestive tract caused by *Candida albicans* (119). Additionally, AhR triggers the IL-22/IL-18 pathway, providing protection against intestinal damage resulting from *vulvovaginal Candidiasis* (120). All of the aforementioned statistics suggest that AhR has the potential to pave the way for antifungal therapy in future medical advancements.

## Conclusions and perspectives

There have been numerous decades of research dedicated to AhR since its discovery in the previous century, and significant progress has been made in understanding the role of AhR in the infection caused by etiological agents. In this review, we have provided an overview of the current knowledge regarding the interaction between AhR’s structural conformations and infections caused by causative agents, as well as the mechanisms of AhR agonists and antagonists in guiding the development of innovative antiviral therapeutic strategies. The AhR receptor primarily mediates the inflammatory response of the respiratory and intestinal barriers when invaded by viruses, bacteria, parasites, and fungi. By upregulating ACE2 expression and regulating tryptophan-associated metabolites like Kyn and IDO, AhR serves as a negative regulator in SARS-CoV-2 infection. The mechanisms by which AhR influences the lung barrier involve the modulation of lymphocytes and neutrophils, leading to the induction of inflammatory cytokines and immune responses during infections caused by influenza virus and other respiratory viruses. Further investigation is required to understand the precise interaction between AhR and immune components. In terms of circadian rhythm regulation, the induction of Bmal1 by miR-10a has been found to decrease the expression of genes related to RA receptors. In addition to the already known circadian genes, further exploration is needed to elucidate the mechanism of rhythm involving additional aryl hydrocarbon receptor nuclear translocators. AhR has been shown to promote tumorigenesis, carcinogenicity, and clonal expansion of transformed liver cells, thereby accelerating the development of hepatocellular carcinoma in individuals with HCV infection. The involvement of LMP1, LMP2A, and the MAPK/ERK pathway in the carcinogenic transformation of EBV is known to attenuate the pro-proliferation effect and nuclear translocation upon AhR inhibition. However, the relationship between AhR pathway and lung carcinoma remains unclear, warranting further investigation in this direction. Additionally, the role of AhR in pathogenic bacterial infections is complex and can have both beneficial and detrimental effects. The positive aspects encompass the protection of the epithelial barrier and cutaneous defense, whereas the negative aspect entails the promotion of chronic inflammation and the consequent long-term bacterial persistence. The overall functionality is contingent upon the bacterial species, cell types, and other yet-to-be-explored factors. AhR primarily governs T cell levels and the secretion of certain cytokines, such as IFN and interleukin, in order to alleviate inflammation during parasitic and fungal infections. Furthermore, the majority of the aforementioned studies primarily focus on singular infections, necessitating a comprehensive exploration of AhR activation in various microbial co-infections. Ultimately, AhR presents a promising therapeutic target for immunomodulation, thereby mitigating the emergence of multi-drug resistance. Enhanced comprehension of the pathogenic mechanisms underlying viral modulation of AhR will facilitate the identification of more effective targets for antiviral therapy.

## Author contributions

LX: Writing – original draft. LL: Funding acquisition, Writing – review & editing. NX: Writing – review & editing, Data curation. WC: Writing – review & editing, Data curation. WN: Writing – review & editing, Funding acquisition, Supervision. RL: Writing – review & editing, Funding acquisition, Supervision.
